# Emotional factors and self-efficacy in the psychological well-being of trainee teachers

**DOI:** 10.3389/fpsyg.2024.1434250

**Published:** 2024-09-10

**Authors:** Raquel Gilar-Corbi, Natalia Perez-Soto, Andrea Izquierdo, Juan-Luis Castejón, Teresa Pozo-Rico

**Affiliations:** Department of Developmental Psychology and Didactics, University of Alicante, Alicante, Spain

**Keywords:** psychological well-being, emotional intelligence, stress, burnout, resilience, self-efficacy, higher education

## Abstract

**Introduction:**

The relationship among emotional intelligence, stress, and self-efficacy is a crucial factor in shaping psychological well-being. It has a significant impact on important areas such as health, academic and professional performance, and overall quality of life.

**Methods:**

Using a hierarchical approach, this study aimed to identify, the specific predictors of psychological well-being, including emotional intelligence, stress, resilience, burnout, and self-efficacy, among higher education students pursuing a bachelor’s degree in education. We also examined gender differences among these predictors. This study involved 338 higher education students pursuing a primary education teaching degree.

**Results:**

The results obtained using the hierarchical regression analysis technique, indicated that the resilience measure, the burnout measure, and the factor of the teacher self-efficacy measure related to self-efficacy in coping with challenges and effectiveness in dealing with change in the educational context, significantly contributed to explaining psychological well-being in the total sample. Furthermore, the predictors of psychological well-being differed between male and female samples.

**Discussion:**

Finally, these findings are discussed in terms of their theoretical and practical implications for improving the training process of future teachers.

## 1 Introduction

In the dynamic and demanding landscape of education, teachers’ psychological well-being is recognized as an essential component of human life that is crucial for effective professional activity and self-efficacy (Tikhomirova et al., 2022). Mugambi et al. (2015) suggested that psychological well-being is crucial not only for teachers’ personal health and satisfaction but also for the quality of the instruction they provide, which influences the overall learning environment. Psychological factors such as emotional intelligence (EI), stress management, resilience, burnout, and self-efficacy are central to this well-being (Kyriazopoulou and Pappa, 2023; Maamari and Salloum, 2023; Mancini et al., 2022; Pozo-Rico et al., 2020; Pozo-Rico et al., 2023; Supervía and Bordás, 2020; Wang and Wang, 2022). Understanding the intricate interplay among these factors is essential for promoting the holistic development and effectiveness of educators. Research has shown that EI is positively associated with psychological well-being and negatively related to burnout among educators (Lucas-Mangas et al., 2022; Suárez-Martel and Santana, 2021). Psychological well-being, particularly environmental mastery and personal growth, mediates the relationship between EI and burnout and acts as a protective factor (Suárez-Martel and Santana, 2021). Occupational stress negatively affects teachers’ psychological well-being, but EI, self-efficacy, coping strategies, and social support can moderate this relationship (Salami, 2010). EI has been found to predict various indicators of well-being as well as physical and psychological health (Sarrionandia and Mikolajczak, 2019). However, methodological limitations in the current research, such as overreliance on self-reported measures and overlap with personality measures, necessitate further investigation to fully understand the intricate interplay between EI, stress management, and educator well-being (Zeidner et al., 2012). Studies such as Akfirat (2020) have shown that general self-efficacy and certain cognitive emotion regulation strategies are significant predictors of psychological well-being in trainee teachers.

The notion of well-being, understood as a reflective appreciation of an individual’s life experiences, has evolved significantly, encompassing perspectives that include subjective, psychological and social well-being, along with holistic views that integrate physical health, mental well-being, community connection, and bonding with the natural environment (Kumar, 2022; Ng and Fisher, 2013). In the educational sphere, teacher well-being has gained new urgency, especially considering the unprecedented challenges posed by the pandemic. The importance of this well-being lies not only in its direct influence on teaching quality but also in its capacity to create positive learning environments and support the socioemotional development and psychological well-being of students. EI, which encompasses the ability to perceive, understand, and regulate emotions (Mayer and Salovey, 1993, 1997), is widely recognized as a cornerstone of adaptive functioning and well-being across various domains. Individuals with high EI are better equipped to navigate interpersonal relationships, cope with stressors, and maintain psychological equilibrium among challenges (Aldrup et al., 2023; Liu et al., 2023; Pozo-Rico et al., 2023). Deepa (2024) found a significant association between EI and psychological well-being among teachers and students in higher education. Razia (2016) also revealed a positive and significant relationship between these variables, although with gender differences, showing that male teachers had better well-being. Conversely, stress responses can manifest as negative emotions, such as anxiety, anger, and depression, which can undermine teacher effectiveness and satisfaction (Igeño-Cano, 2020; Brady et al., 2023; Iacolino et al., 2023). Chronic stress is pervasive in educational settings, significantly influencing teacher well-being and job performance. Lazarus and Folkman’s (1986) Stress and Coping theory provides a framework for understanding how individuals manage perceived stressors, which is vital for educators who face diverse daily challenges. Additionally, chronic stress can lead to burnout, characterized by emotional exhaustion, depersonalization, and reduced personal accomplishment, ultimately undermining educators’ effectiveness and satisfaction (Gutiérrez et al., 2006; Brady et al., 2023; Iacolino et al., 2023). Research has consistently shown a negative relationship between teacher burnout and psychological well-being (Orellana, 2022; Rellon et al., 2024). Other studies have also shown a significant negative relationship between teachers’ occupational stress and their psychological well-being (Leonardi and Astuti, 2023; Vazi et al., 2013; Kaur et al., 2024; Poormahmood et al., 2017). Higher levels of job stress are associated with lower psychological well-being among teachers (Leonardi and Astuti, 2023; Vazi et al., 2013).

In contrast, resilience, originating from the Latin ‘Resilere,’ meaning ‘to bounce back’ or ‘return to the original state,’ is defined as the ability to return to a state of normalcy or even achieve positive change after facing conflict, risk, or failure. Resilient individuals demonstrate the capacity to bounce back from setbacks, adapt to changing circumstances, and maintain a sense of purpose and optimism amid adversity (Rodríguez-Sánchez et al., 2021; Hamadeh et al., 2021; Cheng et al., 2023; Cherkasskyi et al., 2023; Pérez J. M. et al., 2020; Vargas and García, 2021; Saldarriaga et al., 2022). Resilience is associated with teachers’ psychological well-being and has been identified as a significant predictor (Mat-Zin et al., 2023).

Teacher self-efficacy, as defined by Bandura’s (1973) Social Cognitive Theory, refers to a teacher’s belief in their capacity to affect student outcomes. High self-efficacy encourages teachers to set ambitious goals and persist through challenges, thus significantly impacting educational outcomes. Educators with high self-efficacy are more likely to persevere despite challenges and employ effective teaching strategies, which plays a crucial role in shaping teacher motivation, behavior, and job satisfaction (Dunn and Rakes, 2011; Pajares and Schunk, 2002; Lazarides et al., 2021; Lazarides et al., 2023; Aldrup et al., 2023; Geraci et al., 2023; Iacolino et al., 2023). Research has consistently shown a significant negative relationship between teachers’ occupational stress and psychological well-being (Leonardi and Astuti, 2023; Kaur et al., 2024; Poormahmood et al., 2017).

Including EI, stress management, defined as interventions designed to reduce the impact of workplace stressors (Tejasree, 2019), and self-efficacy in teacher training is an essential strategy for promoting an education that transcends academic excellence, seeking the holistic well-being of all members of the educational community (Téllez-Martínez et al., 2021). Focusing on the educational context, the relationship between these three constructs takes on a critical dimension, influencing not only the personal and professional growth of educators but also the creation of optimal learning environments. In this aspect, EI reveals itself as a powerful tool for effectively managing emotions, both one’s own and those of others, offering coping strategies that promote greater self-efficacy and, consequently, reinforced psychological well-being. Recent studies (Navas-Casado et al., 2023) have highlighted its role in improving personal competence perception, emphasizing its contribution to the development of a sense of control and individual efficacy. Given the significance of these psychological constructs in shaping teacher well-being, the objective of this study is to examine their interrelationships and their collective impact on the psychological well-being of higher education students pursuing a bachelor’s degree in education. Specifically, we aim to investigate the predictive power of EI, stress, resilience, burnout, and self-efficacy on psychological well-being while exploring possible gender differences in these predictions.

### 1.1 Emotional intelligence in teacher training

The EI is acknowledged as a key competence in education (Howard and Cogswell, 2018; Pozo-Rico et al., 2018). Mayer and Salovey (1993) defined EI as the ability to manage one’s own emotions and that of others, discern them, and use this information to guide their thoughts and actions. This perspective underscores how EI manifests differently across genders, suggesting that men and women may display variations in their capacities for self-awareness, self-regulation, motivation, empathy, and social skills (Sojer et al., 2024). Research by Akram et al. (2020) and Hassan (2019) indicated a strong correlation between EI and psychological well-being among teachers. Such gender differentiation in EI invites a deeper exploration of how these emotional skills develop and are applied in diverse social and professional contexts, offering a richer understanding of human dynamics (Mercader-Rubio et al., 2022). Although the importance of EI in teacher training has been highlighted, further practical research is needed to develop applied programs and effective methodologies that enable teachers to lead educational projects tailored to school needs, thereby improving the quality of teaching provided (Gilar-Corbi et al., 2019a).

The benefits of EI training for teachers include increased self-esteem, self-efficacy, motivation, and job satisfaction; reduced stress, anxiety, burnout, and conflicts; and improved empathy, assertiveness, cooperation, leadership, creativity, innovation, and teaching quality (Bar-On and Parker, 2000; Procópio et al., 2016). Behavioral approaches to EI suggest that competencies can be developed through training programs in various contexts, with sustainable improvements over time (Gilar-Corbi et al., 2020; Pozo-Rico et al., 2020; Boyatzis, 2008).

Delving the intersection between EI and other domains of personal development, it is essential to direct our attention toward a concept that has gained prominence in recent studies: resilience. The synergistic relationship between EI and the capacity to withstand and overcome adversities represents a fertile field of research that offers promising perspectives. Various studies have found a positive correlation between EI and resilience. Veloso-Besio et al. (2013) and Cebollero-Salinas et al. (2022) both observed that higher levels of EI were linked with greater resilience in special education officers and university students. These findings are consistent with those of González-Valero et al. (2021), who emphasized the crucial role of EI in fostering resilience, especially in adverse situations. This body of research underscores not only the importance of EI as an intrapersonal skill but also its transformative impact on individuals’ ability to face and overcome challenges, thus opening new avenues for the exploration and strengthening of resilience. Resilience, understood as the capacity to react and recover with positive energy despite adversities, is essential in the teaching field to face difficulties, restore balance, and promote personal and professional growth (Aguaded and Almeida, 2016). Its development and strengthening in teachers can be achieved through training in socioemotional competencies, providing skills, strategies, and resources to manage emotions, improve communication, and create a positive and safe learning environment (Gilar-Corbi et al., 2019b; Pozo-Rico et al., 2020).

### 1.2 Stress and burnout in trainee teachers

Adapting to university life is a key period marked by challenges that can impact student well-being and lead to dropout in the first year. Current research underscores the complexity of this transition, highlighting the importance of self-regulation, time management, and coping strategies (Pérez A. B. D. et al., 2020). Self-regulation involves the ability to manage one’s thoughts, emotions, and behaviors in pursuit of specific goals (Kalamazh et al., 2024). Time management is described by Fuente et al. (2015) as being implicitly related to self-regulated learning, functioning as a metacognitive process variable. Furthermore, coping strategies are mechanisms utilized to handle stress, closely linked to self-regulation, underscoring their importance in maintaining psychological resilience (Kalamazh et al., 2024). The conceptualization of academic stress has been clarified, differentiating between stress, anxiety, and worry, and recognizing its multifaceted nature as a challenging interaction between the student and the educational environment (Roa et al., 2023). Teacher stress, defined as an amalgam of negative emotions stemming from challenges such as student discipline, workload, and classroom diversity, is deeply influenced by gender differences (Collie et al., 2012; Liu and Onwuegbuzie, 2012).

Research on gender differences in educators’ psychological well-being revealed complex patterns. Some studies have reported that women have higher overall psychological well-being (Tribhuvan, 2020). Gender differences were observed in several dimensions, including stress levels (Matud et al., 2019), resilience (Baguri et al., 2022), self-efficacy (Wisudawati, 2023), EI (Madhukar, 2021), and burnout (García-Arroyo et al., 2019). These findings highlight the importance of considering gender differences when studying educators’ psychological well-being. Understanding these differences can help educational institutions more effectively address the well-being of faculty members (Nazir et al., 2021).

The incorporation of a gender perspective reveals how social norms and expectations about roles significantly influence teachers’ perceptions and stress management. This approach not only enhances our understanding of the challenges faced by educators but is also fundamental in designing inclusive interventions that promote well-being and equitable professional development. Recent studies underscore the importance of adapting coping strategies and burnout prevention to these gender differences, adapting specific solutions that mitigate stress and foster a healthy and supportive educational environment (Sabater and Mata, 2019; Adasi et al., 2020; Gustems-Carnicer et al., 2020). For instance, a study conducted in Ecuador found that women report higher levels of academic stress perception than men, at a ratio of 2 to 1, highlighting the necessity for differentiated approaches to managing academic stress (Mena et al., 2023). Similarly, another study revealed that during the COVID-19 pandemic, female university students in Spain exhibited significantly higher levels of academic stress than their male counterparts, along with differences in the coping strategies employed by each gender (Marco-Ahulló et al., 2022).

Furthermore, it has been demonstrated that in the educational context, women are more likely to use emotion-focused coping strategies, whereas men tend to favor positive reappraisal and planning strategies to handle academic challenges (Latif and Amirullah, 2020). These differences in coping mechanisms can influence the effectiveness of interventions aimed at reducing stress and preventing burnout in teachers and students. Consider these gender differences is crucial when designing professional development programs and support mechanisms for educators. For example, female teachers often face unique stressors related to work-life balance and societal expectations, necessitating specific interventions to address these needs (Sigcha and Gómez, 2022). Additionally, incorporating a gender lens into professional development programs can help address these specific needs, leading to more effective stress management and improved job satisfaction (Miles and Naumann, 2023). Exams, time management, and academic overload have also emerged in recent studies as key stressors in the university setting (West et al., 2021). These studies suggest that the true source of stress is not the assessments themselves, but rather the combination of a high workload with tight deadlines, emphasizing the relevance of implementing intervention programs that focus on effective time management and the development of productive study habits (Toral et al., 2023). Teacher stress impacts not only the well-being of teachers but also the quality of teaching and academic performance. It also relates to a reduction in teacher self-efficacy (Klassen et al., 2013). Furthermore, it affects the school climate, coexistence, learning, and the development of students, as stressed teachers can transmit negative emotions, generate conflicts, and show hostile or indifferent attitudes (Fernández-Berrocal, 2023).

The relationship between teacher stress and negative aspects, such as decreased job satisfaction, reduced commitment, and increased burnout, continues to be a relevant topic of study (Rojas-Solís et al., 2021). Teacher burnout, understood as a response to chronic work stress, is characterized by emotional exhaustion, depersonalization, and a reduction in personal accomplishment (Maslach and Leiter, 2022). The impact of burnout extends beyond the personal realm of the teacher, negatively affecting the quality of education, generating discontent, dissatisfaction, and an increase in workplace absenteeism. This underscores the importance of effectively addressing this phenomenon to safeguard the integrity and future of the teaching profession (Quintero and Hernández, 2021). Burnout negatively impacts performance and teaching quality, affecting the teacher–student relationship (Vandenberghe and Huberman, 1999; Baek et al., 2023).

Preventing burnout offers protection and contributes to optimal psychological development at work (Braun et al., 2020; McKenzie et al., 2019). Preventing teacher burnout is also associated with classroom benefits, such as reducing disruptive student behavior and enhancing overall stability (Schnaider-Levi et al., 2020; Rana and Soodan, 2019). Moreover, it positively impacts student motivation and academic commitment (Sönmez and Kolaşınlı, 2021; Adams et al., 2019). Teachers with low levels of stress and no symptoms of burnout, coupled with students exhibiting high coping skills, have been shown to have enriched outcomes for students (Wang et al., 2020; Romano et al., 2020).

### 1.3 Teacher self-efficacy and its pivotal role

The importance of psychological well-being has emerged strongly in the training of future teachers, proposing an expansion of educational focus that transcends the traditional emphasis on self-efficacy. Although Bandura (1995) highlighted self-efficacy as a crucial element in individuals’ capacity to manage and execute necessary actions toward achieving specific goals, promoting solid psychological well-being among educators in training has become fundamental. In this initial phase, the consolidation of a robust state of well-being is presented as an essential pillar, particularly when opportunities to validate self-efficacy through practical experiences are limited. Recent supports this comprehensive view of teacher training, which encompasses both the development of self-efficacy and the promotion of psychological well-being, offering a more solid foundation for the professional and personal growth of future educators (Calicchio, 2023). Finally, Hobfoll and Freedy’s (2017) Conservation of Resources Theory (COR) emphasizes the importance of protecting personal resources, such as resilience and self-efficacy, to prevent burnout. Practical applications of this theory are crucial for developing resilience and self-efficacy among educators, fostering an organizational culture that values and supports employee well-being (Eikenhout et al., 2022; Fernández-Valera et al., 2019; Maffoni et al., 2021; Sommovigo et al., 2019). This holistic approach is reinforced by integrating the gender perspective in teacher training, a key element that directly affects self-efficacy and psychological well-being. Research has revealed that psychological well-being, beyond previous experience, is crucial in teacher preparation, reshaping our focus toward job satisfaction, stress, and burnout in the profession (González-Valero et al., 2021). These studies underscore the need for a holistic training approach that includes the gender dimension, not only to enhance perceived self-efficacy but also to promote greater psychological well-being, fostering inclusive and equitable educational practices.

Moreover, a review of studies on ‘teacher self-efficacy’ in various educational contexts sheds light on how this concept influences student motivation and learning outcomes (Firmansyah et al., 2018; Rezaull et al., 2021). Studies similar to those conducted by Bárbara-Sánchez et al. (2021) and Matos et al. (2022) highlight the importance of factors such as satisfaction, preparation, and infrastructure in shaping teacher self-efficacy. These collective contributions highlight the determinative role of teacher self-efficacy in both teaching practices and student outcomes, underscoring its interconnection with addressing psychological well-being and gender perspectives in teacher training to cultivate a healthier and more satisfying educational environment for all.

Recapping current knowledge, it is recognized that teachers’ psychological well-being is crucial not only for their own health and personal satisfaction but also for the quality of the instruction they provide (Tikhomirova et al., 2022). This, in turn, affects the overall learning environment (Mugambi et al., 2015). This well-being is shaped by a number of key psychological factors, including EI (Deepa, 2024), stress (Kaur et al., 2024), resilience (Mat-Zin et al., 2023), burnout (Braun et al., 2020), and self-efficacy (Calicchio, 2023). EI, which is positively associated with psychological well-being and negatively with burnout, plays a protective role (Suárez-Martel and Santana, 2021). The implementation of effective stress management techniques, coupled with the utilization of coping strategies and social support, can serve to mitigate the adverse effects associated with occupational stress (Abad, 2020). The capacity to adapt and overcome adversity, defined as resilience, is also closely linked to teachers’ psychological well-being (Mat-Zin et al., 2023). In terms of self-efficacy, educators who demonstrate a high level of this construct tend to persevere in the face of challenges and implement more effective teaching strategies (Aldrup et al., 2023).

Nevertheless, despite comprehensive research examining these variables in isolation, there is a notable absence of studies that investigate their interrelationship and impact on teachers’ psychological well-being. This lack of emphasis on examining the joint contribution of EI, stress, resilience, burnout, and self-efficacy on psychological well-being represents a significant gap in the existing literature. Gaining insight into the manner in which these factors are intertwined could prove invaluable for the development of more efficacious strategies to promote well-being in educational settings.

The study addresses critical gaps in the existing literature on teachers’ psychological well-being by adopting a comprehensive approach that simultaneously considers EI, resilience, self-efficacy, and burnout. While previous research has often focused on these factors individually or within limited contexts, this study advances the field by systematically exploring the interactions between these elements and their predictive power with respect to the psychological well-being of future educators.

This study makes a significant contribution to the field by developing a more robust theoretical framework that elucidates the interrelationships between EI, resilience, self-efficacy, stress, and burnout, and their collective contribution to explaining the psychological well-being of trainee teachers. This remains an understudied area in the current literature. Moreover, the research enhances our comprehension of the gender-based distinctions in the relationship between these psychological elements and well-being. It offers a nuanced analysis of how the different experiences and challenges faced by male and female educators influence their psychological outcomes.

Moreover, the study proposes intervention strategies based on a comprehensive and multifaceted analysis, offering not only new insights into the interconnections between the concepts under examination, but also a robust foundation for the formulation of training programs and institutional policies that are responsive to the evolving and complex demands of the contemporary educational landscape.

This study aimed to identify key predictors of psychological well-being, such as EI, stress, resilience, burnout, and self-efficacy, among undergraduate students in education, exploring how these factors contribute differently across genders. From this objective, the following hypotheses are derived:

Higher levels of EI are associated with lower levels of stress and burnout and higher levels of resilience, self-efficacy, and psychological well-being.

Conversely, higher levels of stress were associated with increased burnout and decreased psychological well-being, whereas higher levels of resilience and self-efficacy were associated with lower levels of stress. In addition, gender differences may exist in the relationships between these psychological factors and psychological well-being, potentially leading to variations in the predictors of well-being based on gender.

## 2 Materials and methods

### 2.1 Participants

This study included 338 higher education students pursuing a degree in primary education teaching. Among them, 27.2% were female and 72.8% were male, with a mean age of 20.55 (SD = 4.173) years. Moreover, 172 students were in their first year, whereas 166 were in their second year.

### 2.2 Measures

#### 2.2.1 Trait meta-mood scale (TMMS-24)

TMMS-24, developed by Fernández-Berrocal et al. (2004) as a reduced and adapted version of the original scale by Salovey et al. (1995), was used to assess participants’ perceived EI. This scale consists of 24 items and 3 factors: emotional attention (*α* = 0.90), emotional understanding (*α* = 0.90), and emotional regulation (*α* = 0.86). The items were rated on a 5-point Likert scale, from 1 (strongly disagree) to 5 (strongly agree). A representative example is “I can often identify my feelings” (item 10).

#### 2.2.2 Connor–Davidson resilience scale (CD-RISC)

The Spanish-adapted version by Manzano-García and Ayala (2013) of the original scale developed by Connor and Davidson (2003), consisting of 25 items, was used. This version consists of 25 items and 3 components: resilience, cleverness, and optimism. In the study by Pozo-Rico et al. (2023), each component of the scale showed a reliability of α = 0.94 for resilience, α = 0.93 for resourcefulness, and α = 0.92 for optimism. The items were rated on a 5-point Likert scale, from 0 (strongly disagree) to 4 (strongly agree). A representative example is “I tend to bounce back after a hardship or illness” (item 8).

#### 2.2.3 European Spanish version of the perceived stress scale (PSS)

The European Spanish version of a 10-item scale was used to assess participants’ perceived stress, specifically their feelings and thoughts over the last month. Originally developed by Cohen et al. (1983) in the United States, this scale was adapted to the Spanish context by Remor (2006). This adaptation demonstrated a Cronbach alpha of.82. Participants responded on a 5-point Likert scale, ranging from 0 (never), 1 (almost never), 2 (sometimes), 3 (often), to 4 (very often). A representative example is “In the last month, how often have you felt nervous or stressed?” (item 3).

#### 2.2.4 Norwegian teacher self-efficacy scale

The scale by Skaalvik and Skaalvik (2007), comprising 24 items, was used to measure teacher self-efficacy across six dimensions. These include instruction effectiveness (instruction), adaptation of teaching to the individual needs of students (adaptation teaching), achievement of student motivation (achievement motivation), achievement of maintaining discipline in the classroom (maintain discipline), ability to collaborate with members of the educational community (collaborate teachers/parents), and coping with challenges and effectiveness in dealing with change in educational contexts (cope with change). Reliability was demonstrated in the study by Pozo-Rico et al. (2023), with Cronbach alpha values ranging from 0.73 (achievement motivation) to 0.97 (maintain discipline). Participants responded on a 7-point Likert scale, with 1 (not at all sure), 3 (fairly unsure), 5 (fairly sure), and 7 (absolutely sure). An example is “Finding suitable solutions to conflicts of interest that arise with other teachers” (item 7).

#### 2.2.5 Maslach burnout inventory (MBI)

The MBI, consisting of 22 items, was used to explore participants’ feelings and thoughts regarding their work environment through three dimensions: emotional exhaustion, depersonalization, and personal accomplishment. Burnout syndrome is considered present when there are high scores in the first two dimensions (emotional exhaustion and depersonalization) and low scores in the third (personal accomplishment). The Spanish version used has been adapted and validated by Seisdedos (1997), based on the original work of Maslach et al. (1986). The Cronbach alpha values obtained in this validation are as follows: 0.89 for emotional exhaustion, 0.77 for depersonalization, and 0.74 for personal accomplishment. Items were rated on a 7-point Likert scale, from 0 (never) to 6 (every day). A representative example is “At work, I feel that I am ‘at the end of my rope’” (item 20).

#### 2.2.6 Psychological well-being

The Ryff (1989) scale, consisting of 33 items, was used to measure perceived psychological well-being across six factors. Specifically, the Spanish version validated by Pozo-Rico et al. (2023) was used, with the following Cronbach alpha values: 0.91 for self-acceptance, 0.92 for positive relationships, 0.93 for autonomy, 0.91 for environmental mastery, 0.91 for purpose in life, and 0.92 for personal growth. Participants responded on a 6-point Likert scale, from 1 (strongly disagree) to 6 (strongly agree). An example is “I am an active person in carrying out the projects I set for myself” (item 12).

### 2.3 Procedure

The researchers initially sought authorization from professors teaching in the first and second years of the bachelor’s degree to access their classes and propose the study to students. Upon receiving approval, the investigators explained the study objectives to the students and requested their voluntary participation. Compliance with ethical guidelines was ensured, and all participants provided written informed consent following the principles outlined in the Declaration of Helsinki. The investigators administered the measurement instruments during 1-h sessions in the first semester of the 2023–2024 academic year. The study protocol was approved by the Ethics Committee of the University of Alicante (Ref. UA-2021-12-09_2).

### 2.4 Data analysis

From the basic correlational design, a predictive analysis was performed using a hierarchical regression analysis technique to determine whether each block of variables (the different factors of each of the measures used) introduced successively in the regression equation makes a statistically significant additional contribution to psychological well-being beyond that already explained by the previous variables. Finally, hierarchical regression analyses were conducted, differentiating by gender, using the SPSS v.29 software package.

## 3 Results

The results will be presented under different headings depending on the type of data analysis performed.

### 3.1 Descriptive and correlational analyses

The correlation coefficients (Pearson’s r) between the variables are presented in Table 1. Among all the correlations shown in the correlation table, the total score of the psychological well-being measure showed significant positive correlations with two factors of the EI measure (understanding and regulation), all factors of the resilience and teacher self-efficacy measures, and with the personal accomplishment factor of the burnout measure. Simultaneously, it exhibited significant negative correlations with the exhaustion and depersonalization factors of the burnout and stress measures.

**Table 1:** Intercorrelation between variables.

Variable	1	2	3	4	5	6	7	8	9	10	11	128	13	14	15	16	17	18	*M*	*DS*
1.Well-being	1																		173.72	25.26
2.Emotional attention	0.07	1																	31.29	5.74
3.Emotional understanding	0.48**	0.31**	1																28.56	6.34
4.Emotional regulation	0.48**	0.13*	0.48**	1															28.58	6.13
5.Resilience	0.67**	0.05	0.46**	0.55**	1														23.83	5.60
6.Cleverness	0.78**	0.11*	0.50**	0.52**	0.71**	1													21.35	3.81
7.Optimism	0.58**	0.10	0.42**	0.55**	0.72**	−0.65**	1												18.12	3.65
8.PSS	−0.55**	0.24**	−0.35**	−0.45**	−0.54**	−0.56**	−0.44**	1											18.60	6.42
9.Emotional exhaustion	−0.46**	0.12*	−0.28**	−0.28**	−0.31**	−0.31**	−0.26**	0.46**	1										17.40	9.21
10.Depersonalization	−0.22**	−0.06	−0.14**	−0.06	−0.05	−0.08	−0.08	0.11	0.40**	1									2.90	3.61
11.Personal accomplishment	0.26**	0.09	0.18**	0.16**	0.23**	0.19**	0.16**	−0.15**	−0.02	−0.04	1								37.94	10.53
12.Instruction	0.32**	0.20**	0.26**	0.28**	0.27**	0.32**	0.22**	−0.12*	−0.09	−0.07	0.29**	1							24.72	3.67
13.Adaptation teaching	0.35**	0.17**	0.25**	0.29**	0.27**	0.35**	0.25**	−0.13*	−0.14*	−0.10	0.27**	0.88**	1						24.62	3.73
14.Achievement motivation	0.35**	0.16**	0.25**	0.27**	0.28**	0.34**	0.23**	−0.16**	−0.14**	−0.02	0.27**	0.80**	0.82**	1					23.85	3.71
15.Maintain discipline	0.35**	0.14**	0.24**	0.29**	0.32**	0.35**	0.26**	−0.17**	−0.14*	0.02	0.25**	0.79**	0.83**	0.83**	1				23.61	3.88
16.Collaborate teachers/parents	0.34**	0.18**	0.29**	0.31**	0.29**	0.35**	0.27**	−0.17**	−0.13*	−0.05	0.26**	0.81**	0.83**	0.80**	0.82**	1			24.53	3.83
17.Cope with change	0.25**	0.22**	0.20**	0.21**	0.21**	0.29**	0.18**	−0.14*	−0.07	−0.01	0.26**	0.75**	0.78**	0.79**	0.77**	0.73**	1		23.54	3.84
18.Self-efficacy	0.35**	0.20**	0.27**	0.30**	0.30**	0.36**	0.26**	−0.16**	−0.13*	−0.04	0.29**	0.92**	0.94**	0.92**	0.92**	0.91**	0.88**	1	144.88	20.74

*p < 0.05, **p < 0.01.

### 3.2 Hierarchical regression analysis

The results of the cumulative variance partitioning, which was performed through hierarchical regression analysis to predict the psychological well-being of future teachers, are summarized in Table 2. The table includes only the variables found to be significant in the prediction. The entry of the variables has been determined based on the causal sequence derived from the literature reviewed in the introduction. Specifically, the block of variables related to the EI measure was the first to be introduced into the equation (Step1: emotional attention, emotional understanding, emotional regulation). Subsequently, the block of variables related to the resilience measure followed (Step 2: resilience, cleverness, optimism). Next, the stress measure was incorporated (Step 3: stress SPSS). Then, the block of factors related to the self-efficacy measure was introduced (Step 4: instruction, adaptation teaching, achievement motivation, maintain discipline, collaborate teachers/parents, cope with change). Finally, the block of factors related to the burnout measure was added to the analysis (Step 5: emotional exhaustion, depersonalization, personal accomplishment). Each of the blocks made an additional contribution to the explanation of the criterion (psychological well-being). However, although the blocks of variables were initially significant predictors of the criterion variable upon their entry to the equation, some of these variables became non-significant in later steps as other blocks of variables were introduced. Thus, the variables that made a significant contribution to the explanation of psychological well-being were the cleverness factor, followed by the exhaustion factor in a negative direction. In third place was resilience, whereas in fourth place was the coping with challenges and effectiveness in dealing with change in the educational context factor, albeit in a negative direction. The personal fulfillment factor was significant in fifth place, and the depersonalization factor, in a negative direction, was significant in sixth place. Thus, we observed that each block made a statistically significant additional contribution to explaining psychological well-being is statistically significant, resulting in a total explained variance percentage of 71.3%.

**Table 2:** Hierarchical regression analysis predicting psychological well-being: total sample.

Step and predictor variable	*β*	*R*^2^	*R*^2^ change
Step 1		0.316	0.316**
(Constant)			
Emotional understanding	0.346*		
Emotional regulation	0.325*		
Step 2		0.649	0.333**
(Constant)			
Emotional understanding	0.094*		
Resilience	0.190*		
Cleverness	0.578*		
Step 3		0.657	0.009*
(Constant)			
Resilience	0.167*		
Cleverness	0.538*		
Total Stress	−0.129*		
Step 4		0.671	0.014*
(Constant)			
Resilience	0.157*		
Cleverness	0.512*		
Total Stress	−0.150*		
Cope with change	−0.144*		
Step 5		0.713	0.042**
(Constant)			
Resilience	0.143*		
Cleverness	0.523*		
Cope with change	−0.120*		
Emotional exhaustion	−0.172*		
Depersonalization	−0.079*		
Personal accomplishment	0.088*		

*N* = 338. *p < 0.05, **p < 0.001.

Next, hierarchical regression analyses were conducted separately for each gender (male and female), following the same causal sequence as indicated above. The results of these analyses are presented in Tables 3, 4. The findings reveal that, in the female group, each block made an additional contribution to explaining psychological well-being. Specifically, the variables that made a significant contribution to explaining the criterion were as follows: The cleverness factor took first place, followed by the exhaustion factor in a negative direction. In third place was the resilience factor, followed by the self-efficacy factor in the achievement of student motivation. The coping with challenges and effectiveness in dealing with change in the educational context factor took fifth place, albeit in a negative direction. Finally, in sixth place was the personal fulfillment factor. Together, these variables accounted for 73.5% of the total variance explained.

**Table 3:** Hierarchical regression analysis predicting psychological well-being: women.

Step and predictor variable	*β*	*R*^2^	*R*^2^ change
Step 1		0.334	0.334**
(Constant)			
Emotional understanding	0.351*		
Emotional regulation	0.332*		
Step 2		0.678	0.344**
(Constant)			
Emotional understanding	0.096*		
Resilience	0.203*		
Cleverness	0.575*		
Step 3		0.687	0.009*
(Constant)			
Resilience	0.186*		
Cleverness	0.540*		
Total Stress	−0.126*		
Step 4		0.703	0.016
(Constant)			
Resilience	0.174*		
Cleverness	0.510*		
Total Stress	−0.130*		
Achievement motivation	0.154*		
Cope with change	−0.145*		
Step 5		0.735	0.031**
(Constant)			
Resilience	0.162*		
Cleverness	0.521*		
Achievement motivation	0.150*		
Cope with change	−0.136*		
Emotional exhaustion	−0.172*		

*N* = 246. *p < 0.05, **p < 0.001.

**Table 4:** Hierarchical regression analysis predicting psychological well-being: men.

Step and predictor variable	*β*	*R*^2^	*R*^2^ change
Step 1		0.295	0.295**
(Constant)			
Emotional attention	−0.202*		
Emotional understanding	0.323*		
Emotional regulation	0.315*		
Step 2		0.598	0.303**
(Constant)			
Resilience	0.269*		
Cleverness	0.507*		
Step 3		0.631	0.033*
(Constant)			
Cleverness	0.382*		
Total Stress	−0.284*		
Step 4		0.655	0.023
(Constant)			
Cleverness	0.335*		
Total Stress	−0.330*		
Step 5		0.733	0.079**
(Constant)			
Cleverness	0.394*		
Depersonalization	−0.205*		
Personal accomplishment	0.161*		

*N* = 92. *p < 0.05, **p < 0.001.

In contrast, in the male group, the results of the hierarchical regression indicate that each block made an additional contribution to explaining psychological well-being. In addition, the variables that made a significant contribution to the explanation of the criterion were as follows: the cleverness factor took first place, followed by the depersonalization factor, albeit the negative sense. Finally, in third place was the personal fulfillment factor. Together, these variables accounted for 73.3% of the total variance explained.

## 4 Discussion

The findings of this study offer a thorough examination of the intricate relationships among EI, resilience, burnout, teacher self-efficacy, and psychological well-being in prospective teachers. They present a complex web of interconnected factors that shape the mental landscape of future educators. In today’s dynamic and ever-evolving educational environment, where teachers face diverse challenges on a daily basis, understanding the elements contributing to their psychological well-being is crucial, consistent with other studies highlighting the importance of this issue (Lucas-Mangas et al., 2022; Pei et al., 2022; Pozo-Rico et al., 2023). This study not only reveals the complex interplay among these factors but also provides insight into the nuanced nature of psychological well-being within the context of teacher training.

First, the correlation analysis revealed significant associations among the various psychological constructs measured in the study. Notably, psychological well-being exhibited positive correlations with factors of EI, resilience, teacher self-efficacy, and personal accomplishment, whereas it showed negative correlations with burnout factors and stress. These findings align with previous research indicating the interconnectedness of these constructs within the context of teacher well-being (Liang et al., 2022; Mennes et al., 2023). Specifically, factors such as emotional regulation, resilience, and self-efficacy appear to play pivotal roles in shaping teachers’ psychological well-being (Carroll et al., 2021; Ghasemi, 2022; Mclean et al., 2023a; Yang and Du, 2024). Furthermore, the differential correlations observed among the variables underscore the nuanced interplay between individual characteristics and environmental stressors in influencing teacher mental health (Derakhshan et al., 2023; Yang et al., 2023).

Second, the hierarchical regression analysis further elucidated the unique contributions of different psychological factors to predicting psychological well-being among future teachers, consistent with findings from previous studies (Branquinho et al., 2023; Zhang and Luo, 2023). The sequential entry of variables based on theoretical frameworks revealed the cumulative impact of EI, resilience, stress, self-efficacy, and burnout on well-being, in line with other research findings (Baguri et al., 2022; Ratanasiripong et al., 2022; Zhang and Luo, 2023). Notably, resilience emerged as a consistent predictor across genders. However, gender-specific analyses revealed nuanced patterns: women exhibited stronger associations between self-efficacy and well-being, whereas men demonstrated heightened sensitivity to depersonalization within the burnout construct. These gender-specific nuances underscore the importance of considering diverse individual characteristics and experiences in promoting teacher well-being.

It is important to note that when the variables related to EI were introduced in the first step of the hierarchical regression, they significantly contributed to the explanation/prediction of the criterion variable, psychological well-being. However, when the variables related to resilience were included in the equation in the second step, some of the variables related to EI no longer made a significant contribution. In addition, when the variable related to stress was introduced in the third step, EI no longer significantly contributed to explaining the criterion variable. Similarly, the stress variable, when introduced in the third step, significantly contributed to the explaining the criterion variable. However, in the fifth step, when the burnout variables were introduced, stress no longer significantly contributed to the prediction or explanation of psychological well-being.

Considering that each block of variables independently has a significant effect on psychological well-being, the combined effect of all variables diminishes the significance of EI and stress. This indicates that EI and stress may have an indirect effect on psychological well-being, possibly mediated by resilience and burnout. As a topic for future research, we suggest further investigation into the moderating and mediating relationships among the study variables.

Finally, the findings not only highlight the multifaceted nature of teacher well-being but also underscore the intricate interplay of psychological factors that influence it. Teachers play a pivotal role in shaping the academic, emotional, and social development of their students. However, they often encounter significant challenges and stressors within the educational landscape (Altinay and Bicentürk, 2023). As evidenced by the results, factors such as resilience, EI, self-efficacy, and burnout collectively impact teacher well-being, reflecting a complex web of individual characteristics and environmental pressures.

To cultivate a nurturing and sustainable educational environment, it could be beneficial for educators to implement targeted strategies aimed at bolstering key psychological factors. Employing such approaches may be advisable to ensure the well-being and effectiveness of the teaching staff (Agyapong et al., 2023; Emerson et al., 2023). Enhancing teacher resilience could be crucial, as it equips teachers with the adaptive coping skills necessary to navigate the myriad challenges inherent in the teaching profession. Resilient teachers might be better equipped to bounce back from setbacks, maintain a positive outlook despite adversity, and maintain their passion for teaching despite the inherent stressors (Abu Hasan et al., 2022; Emerson et al., 2023; Han et al., 2023; Mao et al., 2024).

### 4.1 Educational implications and future research directions

A key practical implication could be the development of specific training programs that offer concrete tools and strategies to strengthen resilience, as it is rarely included as a specific training topic (Regalado et al., 2021). Enhancing the capacity of trainee teachers to face daily classroom challenges in the future may be essential, as evidenced by other studies, such as that of Bordás (2023). These programs could include mindfulness workshops and stress management techniques designed to provide educators with effective resources to manage stress and meet students’ diverse needs (Haydon et al., 2019; Terrell et al., 2023).

Moreover, fostering EI among teachers may promote self-awareness, empathy, and effective interpersonal relationships. By cultivating EI skills, educators could better understand and manage their own emotions, navigate interpersonal dynamics with students and colleagues, and create a supportive classroom environment conducive to learning and well-being (Maharaj and Ramsaroop, 2022; Wang, 2023; Woolcott et al., 2023).

In addition, empowering teachers with a strong sense of self-efficacy could be crucial for bolstering their confidence in their ability to positively impact student outcomes. Teachers with high self-efficacy beliefs are more likely to set ambitious goals, persist in the face of challenges, and use effective teaching strategies to meet students’ diverse needs (Chen and Liu, 2020; Wilcoxen et al., 2020). Providing professional development opportunities that enhance teacher efficacy could have far-reaching benefits not only for teacher well-being, but also for student achievement and overall school climate (Karim et al., 2022).

A practical strategy to achieve this might be to establish peer support networks in which active professionals at the same educational stage as trainee teachers can share experiences and strategies for managing situations of high emotional demand in the classroom. These networks could allow trainee teachers to analyze real situations and develop possible responses, taking into account the experiences and suggestions of their colleagues. Descoeudres (2023) suggest that sharing experiences might foster wellbeing and professional development. This could facilitate the acquisition of skills to resolve conflicts with students or colleagues in a respectful and effective manner, thereby promoting a collaborative and continuous learning environment. For example, Boyle (2019) emphasized the importance of developing a professional support network that includes direct supervisors, colleagues, and mentors to manage mental health and gain deeper insights into teaching.

Furthermore, mitigating burnout among teachers could be paramount to preserving their mental health and preventing attrition within the profession. Burnout not only diminishes teachers’ well-being but can also compromise their effectiveness in the classroom and detract from student-learning outcomes (Burger, 2024; Cohen et al., 2024; Igu et al., 2023; Mclean et al., 2023b). Implementing organizational policies and practices that promote work–life balance, foster a culture of appreciation and support, and provide resources for stress management and emotional regulation could help mitigate burnout risk among educators (Nemiro et al., 2022; Ulla and Poom-Valickis, 2023).

Moreover, recognizing gender-specific differences in the manifestation and predictors of teacher well-being underscores the importance of tailoring interventions to address the unique needs and challenges faced by male and female educators. For example, interventions targeting self-efficacy might be particularly beneficial for female teachers who may face additional societal pressures and expectations in balancing their professional and personal roles. Conversely, interventions aimed at mitigating depersonalization and fostering a sense of personal fulfillment might be more salient for male teachers, who may experience distinct challenges in navigating their professional identities and emotional well-being within the educational context (García-Alvarez et al., 2023; Kollerová et al., 2023; Masková, 2023; Seibt and Kreuzfeld, 2023).

Essentially, fostering teacher well-being may require a multifaceted approach that addresses the unique interplay of psychological factors while recognizing and addressing gender-specific differences. By investing in resilience, EI, self-efficacy, and burnout-mitigation strategies tailored to the diverse needs of educators, we could create a more supportive and sustainable educational environment that nurtures both teacher and student success.

Lastly, the limitations of this study are worth noting, as they may have influenced the results. Firstly, the sample was limited to 338 primary education students from a single institution. This restriction might limit the generalizability of the findings to other student populations in different geographical and educational contexts. Future research could benefit from considering larger and more diverse samples to verify the external validity of the obtained results.

Moreover, the cross-sectional design of the study prevents the establishment of causal relationships between the examined variables. Although significant associations were identified, it cannot be concluded that one variable causes changes in another. Longitudinal studies might be necessary to examine how these relationships develop and change over time, providing a deeper and more dynamic understanding of the factors influencing the psychological well-being of trainee teachers.

Another important limitation is the use of self-report questionnaires for data collection, which can introduce biases such as social desirability and a lack of accurate introspection by participants. Future research could benefit from incorporating mixed methods, including qualitative interviews and direct observations, to complement and validate the quantitative data and offer a more holistic view of teachers’ experiences.

The study focused on a specific set of variables: EI, stress, resilience, burnout, and self-efficacy. However, other important factors, such as social support, intrinsic motivation, and working conditions, were not considered. Future research might explore the influence of these and other additional variables to obtain a more comprehensive understanding of psychological well-being in teachers.

As for future research directions, it could be highly beneficial to develop and evaluate intervention programs designed to improve resilience and EI among students and teachers, given the relevance of these factors in psychological well-being identified in the present study. Evaluating the long-term effectiveness of these programs and their impact on well-being and professional performance might provide valuable practical contributions.

It would also be pertinent to investigate the possible mediating and moderating roles of resilience and burnout in the relationship between EI and psychological well-being. This may provide a more detailed view of the underlying mechanisms that influence well-being, allowing for the development of more specific and effective interventions.

Additionally, exploring how different educational contexts and institutional policies affect the psychological well-being of teachers might offer important practical implications. Comparing institutions with different levels of organizational support and resources might allow for the identification of effective practices that could be more widely adopted.

Finally, conducting longitudinal studies that follow education students throughout their training and into the early years of their professional careers could help understand how well-being factors and coping strategies evolve over time. Similarly, examining how cultural differences can influence the relationships between EI, stress, resilience, self-efficacy, and psychological well-being through comparative studies between different countries could reveal significant cultural variations and similarities.

Incorporating these considerations and suggestions in future research could strengthen the field of study by providing a more complete and nuanced understanding of psychological well-being in the context of teacher training.

### 4.2 Translating research into practice

Building on the insights gained from this study, it may be beneficial to develop practical applications that directly address educators’ needs and challenges. Specifically, the following actions may be prioritized:

1. Implementation of Resilience Training: based on the study’s findings, creating targeted resilience training programs for teachers may be valuable. These programs could focus on developing coping strategies, stress management techniques, and enhancing emotional resilience to better handle the demands of the teaching profession (Abu Hasan et al., 2022; Regalado et al., 2021).
2. Promotion of EI: integrating EI development into professional development curricula may help teachers manage their emotions and improve their interpersonal relationships. This could contribute to a more supportive and effective teaching environment (Maharaj and Ramsaroop, 2022; Wang, 2023).
3. Support for Self-Efficacy: enhancing teachers’ self-efficacy through targeted professional development opportunities could be crucial. This may involve workshops and mentoring programs designed to build confidence and improve teaching effectiveness (Chen and Liu, 2020; Karim et al., 2022).
4. Addressing Burnout: implementing organizational practices that promote work-life balance and provide stress management resources could be instrumental in mitigating burnout and supporting teacher well-being (Burger, 2024; Nemiro et al., 2022).

By focusing on these practical applications, educational institutions could more effectively address the psychological factors highlighted in this study, fostering a healthier and more supportive environment for both teachers and students.

Our research makes a substantial contribution to the field of teacher well-being by exploring the intricate interplay between EI, resilience, self-efficacy, stress and burnout. Whereas previous studies have often investigated these factors in isolation, our study integrates them into a unified theoretical framework. This approach provides a more comprehensive understanding of the interrelationships between these elements and their predictive power with regard to teacher well-being, addressing an important gap in the current literature. By bringing these factors together in a coherent model, we advance our understanding of the collective impact of these variables on educators’ psychological well-being and contribute to the development of a more comprehensive theoretical framework.

In addition to enhancing theoretical comprehension, our research provides significant insights into the gender-specific nuances of the interrelationships between these psychological variables and psychological well-being. This nuanced perspective helps to highlight the varied experiences and challenges faced by male and female educators, emphasizing the need for gender-sensitive approaches in developing interventions. Such insights are crucial for designing more effective and personalized strategies that cater to the diverse needs of teachers.

The study also provides actionable recommendations for translating these findings into practice. Specifically, we suggest developing targeted resilience training programs to equip teachers with coping strategies and stress management techniques. Incorporating EI development into professional training curricula can further support teachers in managing their emotions and improving interpersonal relationships. Enhancing self-efficacy through workshops and mentoring programs can build teachers’ confidence and effectiveness. Additionally, implementing organizational practices that promote work-life balance and provide stress management resources can help mitigate burnout. These practical recommendations aim to foster a supportive and healthy educational environment, benefiting both teachers and students.

## 5 Conclusion

The findings of this research underscore the intricate web of psychological factors that influence teacher well-being and how these elements directly impact the quality and sustainability of the educational environment. Our commitment to educational excellence and quality drives us to recognize the fundamental importance of bolstering resilience, EI, and self-efficacy among educators while mitigating burnout.

Targeted and effective intervention strategies could be beneficial in addressing these challenges and moving toward a more robust education system suitable for the 21st century. First, we could invest in professional development programs that strengthen teachers’ resilience, EI, and self-efficacy. These programs could provide educators with the tools and resources needed to navigate the complexities of their profession and thrive amid adversity (Bishop and High, 2023; Pozo-Rico and Sandoval, 2020; Romano et al., 2021; Sousa and Barros, 2017; Wilkins et al., 2023).

Furthermore, organizational policies and practices could be implemented to create a supportive and nurturing work environment. This includes promoting work–life balance, fostering a culture of appreciation and support, and providing resources for stress management and emotional well-being. By prioritizing teacher well-being, we can cultivate a more positive and enriching educational experience for both teachers and students alike (Li et al., 2022; Liang, 2024; Suleyeva et al., 2022).

Moving forward, it might be beneficial to tailor interventions to address the unique needs and challenges faced by educators, while also recognizing and embracing the evolving demands of the 21st century. Therefore, it could be advantageous to employ strategies that promote innovation, diversity, and adaptability in a dynamic educational landscape. Emphasizing these principles could help maintain responsiveness to the dynamic nature of modern education, ultimately fostering an environment conducive to growth and success for both educators and students (García-Alvarez et al., 2023; Kollerová et al., 2023; Masková, 2023; Seibt and Kreuzfeld, 2023).

## Data Availability

The raw data supporting the conclusions of this article will be made available by the authors, without undue reservation.

## References

[ref1] Abad S. M. (2020). El estrés, sus consecuencias y cómo afrontarlo. Nuberos Científica 4, 31–36.

[ref2] Abu Hasan R. Yusoff M. S. B. Tang T. B. Hafeez Y. Mustafa M. C. Dzainudin M.. (2022). Resilience-building for mental health among early childhood educators: a systematic review and pilot-study towards an EEG-VR resilience building intervention. Int. J. Environ. Res. Public Health 19:4413. doi: 10.3390/Ijerph19074413, PMID: 35410097 PMC8998227

[ref3] Adams K. Lumb A. Tapp J. Paige R. (2019). Whole child, whole teacher: leadership for flourishing primary schools. Education 48, 861–874. doi: 10.1080/03004279.2019.1666419

[ref4] Adasi G. S. Amponsah K. D. Mohammed S. M. Yeboah R. Mintah P. C. (2020). Gender differences in stressors and coping strategies among teacher education students at University of Ghana. J. Educ. Learn. 9, 123–133. doi: 10.5539/jel.v9n2p123

[ref5] Aguaded M. C. Almeida N. A. (2016). The resilience of teachers as crucial factor to overcome adversity in a society of changes. Tendencias Pedagógicas 28, 167–180. doi: 10.15366/tp2016.28.012

[ref6] Agyapong B. Chishimba C. Wei Y. Dias R. Eboreime E. Msidi E.. (2023). Improving mental health literacy and reducing psychological problems among teachers in Zambia: protocol for implementation and evaluation of a Wellness4Teachers email messaging program. JMIR Research Protocols 12:e44370. doi: 10.2196/44370, PMID: 36877571 PMC10028515

[ref7] Akfirat O. N. (2020). Investigation of relationship between psychological well-being, self esteem, perceived general self-efficacy, level of hope and cognitive emotion regulation strategies. Euro. J. Educ. Stud. 7, 286–306. doi: 10.46827/EJES.V7I9.3267

[ref8] Akram M. Munir F. Gilani M. (2020). Relationship between emotional intelligence and psychological well-being of secondary school teachers. Global Educ. Studies Rev. 5, 108–121. doi: 10.31703/gesr.2020(V-IV).12

[ref9] Aldrup K. Carstensen B. Klusmann U. (2023). The role of teachers' emotion regulation in teaching effectiveness: a systematic review integrating four lines of research. Educ. Psychol. 59:446. doi: 10.1080/00461520.2023.2282446

[ref10] Altinay Z. Bicentürk B. (2023). Constructing sustainable learning ecology to overcome burnout of teachers: perspective of organizational identity and locus of control. Sustain. For. 15:16930. doi: 10.3390/su152416930

[ref11] Baek S. U. Yoon J. H. Won J. U. (2023). Association between high emotional demand at work, burnout symptoms, and sleep disturbance among Korean workers: a cross-sectional mediation analysis. Sci. Rep. 13:16688. doi: 10.1038/s41598-023-43451-w 37794088 PMC10550909

[ref12] Baguri E. M. Roslan S. Hassan S. A. Krauss S. E. Zaremohzzabieh Z. (2022). How do self-esteem, dispositional hope, crisis self-efficacy, mattering, and gender differences affect teacher resilience during COVID-19 school closures? Int. J. Environ. Res. Public Health 19:4150. doi: 10.3390/ijerph19074150, PMID: 35409829 PMC8998510

[ref13] Bandura A. (1973). Aggression: A social learning analysis. EEUU: Prentice-Hall.

[ref14] Bandura A. (1995). “Exercise of personal and collective efficacy in changing societies” in Self-efficacy in changing societies. ed. Bandura A. (New York: Cambridge University Press).

[ref15] Bárbara-Sánchez V. Orozco-Barbosa L. Arias-Antúnez E. (2021). On the impact of information technologies secondary-school capacity in business development: evidence from smart cities around the world. Front. Psychol. 12:731443. doi: 10.3389/fpsyg.2021.731443, PMID: 34970182 PMC8712570

[ref16] Bar-On R. E. Parker J. D. (2000). The handbook of emotional intelligence: Theory, development, assessment, and application at home, school, and in the workplace. San Francisco: Jossey-Bass.

[ref17] Bishop R. E. High A. C. (2023). Caregiving in academia: examining educator well-being and burnout during prolonged stressors. Pers. Relationships 30, 1274–1292. doi: 10.1111/pere.12513

[ref18] Bordás A. (2023). Investigation of resilience and social mobility in education. Central Euro. J. Educ. Res. 5, 24–36. doi: 10.37441/cejer/2023/5/1/11119, PMID: 38347746

[ref19] Boyatzis R. E. (2008). Leadership development from a complexity perspective. Consulting Psychol. J.: Pract Res. 60, 298–313. doi: 10.1037/1065-9293.60.4.298, PMID: 39207245

[ref20] Boyle D. (2019). “Looking after yourself and your professional development” in Surviving and thriving in the secondary school. eds. Capel S. Lawrence J. Leask M. Younie S. (London: Routledge).

[ref21] Brady L. L. McDaniel S. C. Choi Y. J. (2023). Teacher stress and burnout: the role of psychological work resources and implications for practitioners. Psychol. Sch. 60, 1706–1726. doi: 10.1002/pits.22805

[ref22] Branquinho C. Moraes B. Noronha C. Ferreira T. Rodrigues N. N. de Matos M. G. (2023). Perceived quality of life and life satisfaction: does the role of gender, age, skills, and psychological factors remain relevant after the COVID-19 pandemic? Children 10:1460. doi: 10.3390/children10091460, PMID: 37761421 PMC10528662

[ref23] Braun S. S. Schonert-Reichl K. A. Roeser R. W. (2020). Effects of teachers’ emotion regulation, burnout, and life satisfaction on student well-being. J. App. Dev. Psychol. 69:101151. doi: 10.1016/j.appdev.2020.101151

[ref24] Burger J. (2024). Constructivist and transmissive mentoring: effects on teacher self-efficacy, emotional management, and the role of novices’ initial beliefs. J. Teacher Educ. 75, 107–121. doi: 10.1177/00224871231185371

[ref25] Calicchio S. (2023). Albert Bandura y el factor de autoeficacia: Un viaje a la psicología del potencial humano a través de la comprensión y el desarrollo de la autoeficacia y la autoestima. Madrid: Stefano Calicchio.

[ref26] Carroll A. York A. Fynes-Clinton S. Sanders-O'Connor E. Flynn L. Bower J. M.. (2021). The downstream effects of teacher well-being programs: improvements in teachers' stress, cognition and well-being benefit their students. Front. Psychol. 12:689628. doi: 10.3389/fpsyg.2021.689628, PMID: 34276519 PMC8281271

[ref27] Cebollero-Salinas A. Bautista P. Cano J. Orejudo S. (2022). E-competencias e inteligencia colectiva. Propuestas para el desarrollo emocional en las interacciones en línea. Rev. Int. Educ. Emoc. Bien. 2, 13–32. doi: 10.48102/rieeb.2022.2.1.21

[ref28] Chen S. Y. Liu S. Y. (2020). Developing students’ action competence for a sustainable future: a review of educational research. Sustain. For. 12:1374. doi: 10.3390/su12041374

[ref29] Cheng H. Fan Y. Q. Lau H. (2023). An integrative review on job burnout among teachers in China: implications for human resource management. Int. J. Human Resource Manag. 34, 529–561. doi: 10.1080/09585192.2022.2078991

[ref30] Cherkasskyi A. Lapchenko I. Meloian A. Kiian A. Pohribna A. (2023). Leader's influence on the climate in the team. Rev. Amaz. Inv. 12, 216–226. doi: 10.34069/ai/2023.69.09.19

[ref31] Cohen S. Kamarck T. Mermelstein R. (1983). A global measure of perceived stress. J. Health Social Behav. 24, 385–396. doi: 10.2307/2136404, PMID: 6668417

[ref32] Cohen J. Wong V. C. Krishnamachari A. Erickson S. (2024). Experimental evidence on the robustness of coaching supports in teacher education. Educ. Res. 53, 19–35. doi: 10.3102/0013189X231198827

[ref33] Collie R. J. Shapka J. D. Perry N. E. (2012). School climate and social-emotional learning: predicting teacher stress, job satisfaction, and teaching efficacy. J. Educ. Psychol. 104, 1189–1204. doi: 10.1037/a0029356

[ref34] Connor K. M. Davidson J. R. (2003). Development of a new resilience scale: the Connor-Davidson resilience scale (CD-RISC). Depres. Anxiety 18, 76–82. doi: 10.1002/da.10113, PMID: 12964174

[ref35] Deepa R. (2024). Impact of emotional intelligence on the well-being of teachers and students. Qeios:1, 1–13. doi: 10.32388/5RY3YW

[ref36] Derakhshan A. Greenier V. Fathi J. (2023). Exploring the interplay between a loving pedagogy, creativity, and work engagement among EFL/ESL teachers: a multinational study. Curr. Psychol. 42, 22803–22822. doi: 10.1007/s12144-022-03371-w

[ref37] Descoeudres M. (2023). The collective dimension in the activity of physical education student-teachers to cope with emotionally significant situations. Educ. Sciences 13:437. doi: 10.3390/educsci13050437

[ref38] Dunn K. E. Rakes G. C. (2011). Teaching teachers: an investigation of beliefs in teacher education students. Learn. Environ. Res. 14, 39–58. doi: 10.1007/s10984-011-9083-1, PMID: 39020214

[ref39] Eikenhout L. M. J. Delahaij R. Van Dam K. Kamphuis W. Hulshof I. L. Van Ruysseveldt J. (2022). Chronic stressors and burnout in Dutch police officers: two studies into the complex role of coping self-efficacy. Front. Psychol. 13:1054053. doi: 10.3389/fpsyg.2022.1054053 36591030 PMC9800995

[ref40] Emerson D. J. Hair J. F. Smith K. J. (2023). Psychological distress, burnout, and business student turnover: the role of resilience as a coping mechanism. Res. Higher Educ. 64, 228–259. doi: 10.1007/s11162-022-09704-9, PMID: 35789581 PMC9243806

[ref41] Fernández-Berrocal P. (2023). Inteligencia emocional: Aprender a gestionar las emociones. Barcelona: Shackleton Books.

[ref42] Fernández-Berrocal P. Extremera N. Ramos N. (2004). Validity and reliability of the Spanish modified version of the trait meta-mood scale. Psychol. Reports 94, 751–755. doi: 10.2466/pr0.94.3.751-755, PMID: 15217021

[ref43] Fernández-Valera M. M. Soler-Sánchez M. I. García-Izquierdo M. Meseguer de Pedro M. (2019). Personal psychological resources, resilience and self-efficacy and their relationship with psychological distress in situations of unemployment. Int. J. Soc. Psychol. 34, 331–353. doi: 10.1080/02134748.2019.1583513

[ref44] Firmansyah F. Komala R. Rusdi R. (2018). Self-efficacy and motivation: improving biology learning outcomes of senior high school students. J. Pendidikan Biologi Indonesia 4, 203–208. doi: 10.22219/jpbi.v4i3.6878, PMID: 27409075

[ref45] Fuente J. Zapata L. Martínez-Vicente J. M. Sander P. Putwain D. (2015). “Personal self-regulation, self-regulated learning and coping strategies, in university context with stress” in Metacognition: fundaments, applications, and trends. intelligent systems reference library, Ed. A. Peña-Ayala (Cham: Springer), 223–255.

[ref46] García-Alvarez D. Soler M. J. Cobo-Rendón R. Hernández-Lalinde J. (2023). Teacher professional development, character education, and well-being: multicomponent intervention based on positive psychology. Sustain. For. 15:9852. doi: 10.3390/su15139852

[ref47] García-Arroyo J. A. Osca Segovia A. Peiró J. M. (2019). Meta-analytical review of teacher burnout across 36 societies: the role of national learning assessments and gender egalitarianism. Psychol. Health 34, 733–753. doi: 10.1080/08870446.2019.1568013, PMID: 30688087

[ref48] Geraci A. Di Domenico L. Inguglia C. D'Amico A. (2023). Teachers' emotional intelligence, burnout, work engagement, and self-efficacy during COVID-19 lockdown. Behav. Sci. 13:296. doi: 10.3390/bs13040296, PMID: 37102810 PMC10135634

[ref49] Ghasemi F. (2022). The effects of dysfunctional workplace behavior on teacher emotional exhaustion: a moderated mediation model of perceived social support and anxiety. Psychol. Reports 16:699. doi: 10.1177/00332941221146699 36527284

[ref50] Gilar-Corbi R. Pozo-Rico T. Castejón J.-L. (2019b). Desarrollando la inteligencia emocional en educación superior: Evaluación de la efectividad de un programa en tres países. Educ. 22, 161–187. doi: 10.5944/educXX1.19880

[ref51] Gilar-Corbi R. Pozo-Rico T. Pertegal-Felices M. L. Sanchez B. (2019a). Emotional intelligence training intervention among trainee teachers: a quasi-experimental study. Psicol. Reflex. Crít. 31:33. doi: 10.1186/S41155-018-0112-1 PMC6967020 32026146

[ref52] Gilar-Corbi R. Pozo-Rico T., Castejón, J-L., Sánchez T. Sandoval-Palis I. Vidal J. (2020). Academic achievement and failure in university studies: motivational and emotional factors. Sustain. For. 12:9798. doi: 10.3390/su12239798, PMID: 38758174

[ref53] González-Valero G. Zurita-Ortega F. San Román-Mata S. Puertas-Molero P. (2021). Relación de efecto del Síndrome de Burnout y resiliencia con factores implícitos en la profesión docente: una revisión sistemática. Rev. Educ. 394:508. doi: 10.4438/1988-592X-RE-2021-394-508

[ref54] Gustems-Carnicer J. Calderon C. Calderon-Garrido D. Martin-Piñol C. (2020). Academic progress, coping strategies and psychological distress among teacher education students. Int. J. Educ. Psychol. 9, 290–312. doi: 10.17583/ijep.2020.4905

[ref55] Gutiérrez G. A. Celis M. Á. C. Moreno S. Farías F. de Suárez J. (2006). Síndrome de burnout. Arch. Neurociencias 11, 305–309.

[ref56] Hamadeh S. Garvey L. Willetts G. Olasoji M. (2021). Undergraduate nursing students’ resilience, challenges, and supports during corona virus pandemic. Int. J. Mental Health Nursing 30, 1407–1416. doi: 10.1111/inm.12896, PMID: 34109714

[ref57] Han F. L. Duan R. R. Huang B. B. Wang Q. L. (2023). Psychological resilience and cognitive reappraisal mediate the effects of coping style on the mental health of children. Front. Psychol. 14:1110642. doi: 10.3389/Fpsyg.2023.1110642 37077843 PMC10106575

[ref58] Hassan M. (2019). Emotional intelligence and subjective wellbeing: exploration of teachers’ burning dilemma. Problems Psychol. 13:101. doi: 10.33225/ppc/19.13.101

[ref59] Haydon T. Alter P. Hawkins R. Theado C. K. (2019). “Chack yourself”: mindfulness-based stress reduction for teachers of students with challenging behaviors. Beyond Behav. 28, 55–60. doi: 10.1177/1074295619831620, PMID: 37174145

[ref60] Hobfoll S. E. Freedy J. (2017). “Conservation of resources: a general stress theory applied to burnout” in Professional burnout. eds. Schaufeli W. B. Maslach C. Marek T. (London: Routledge), 115–129.

[ref61] Howard M. C. Cogswell J. E. (2018). The “other” relationships of self-assessed intelligence: a meta-analysis. J. Res. Pers. 77, 31–46. doi: 10.1016/j.jrp.2018.09.006

[ref62] Iacolino C. Cervellione B. Isgrò R. Lombardo E. M. C. Ferracane G. Barattucci M.. (2023). The role of emotional intelligence and metacognition in teachers' stress during pandemic remote working: a moderated mediation model. Euro. J. Inv. Health Psychol. Educ. 13, 81–95. doi: 10.3390/ejihpe13010006, PMID: 36661756 PMC9857521

[ref63] Igeño-Cano J. C. (2020). Beneficios de los paseos por jardines exteriores del hospital en el paciente crítico, familia y profesionales. Med. Intensiva 44, 446–448. doi: 10.1016/j.medin.2019.09.007, PMID: 31690465

[ref64] Igu N. C. N. Ogba F. N. Eze U. N. Binuomote M. O. Elom C. O. Nwinyinya E.. (2023). Effectiveness of cognitive behavioral therapy with yoga in reducing job stress among university lecturers. Front. Psychol. 13:950969. doi: 10.3389/fpsyg.2022.950969, PMID: 36687866 PMC9849775

[ref65] Kalamazh V. Tymoshchuk E. Krasnopir A. Doroshchuk A. (2024). Coping strategies as a component of self-regulation of students' educational activities. Scient Notes Ostroh Acad. Nat. Univ. Psychol Series 1, 28–35. doi: 10.25264/2415-7384-2023-16-28-35

[ref67] Karim N. Othman H. Zaini Z. I. I. Rosli Y. Wahab M. I. A. Al Kanta A.. (2022). Climate change and environmental education: stance from science teachers. Sustain. For. 14:16618. doi: 10.3390/su142416618

[ref68] Kaur M. Khosla R. Siddiqui M. (2024). Impact of job stress on psychological well-being of teachers. Int. Res. J. Adv. Eng. Manag. 2, 504–515. doi: 10.47392/IRJAEM.2024.0071

[ref69] Klassen R. Wilson E. Siu A. F. Y. Hannok W. Wong M. W. Wongsri N.. (2013). Preservice teachers’ work stress, self-efficacy, and occupational commitment in four countries. Euro. J. Psychol. Educ. 28, 1289–1309. doi: 10.1007/s10212-012-0166-x

[ref70] Kollerová L. Kveton P. Zábrodská K. Janosová P. (2023). Teacher exhaustion: the effects of disruptive student behaviors, victimization by workplace bullying, and social support from colleagues. Social Psychol. Educ. 26, 885–902. doi: 10.1007/s11218-023-09779-x

[ref71] Kumar N. (2022). Mental health and well-being: an Indian psychology perspective. London: Routledge India.

[ref72] Kyriazopoulou M. Pappa S. (2023). Emotional intelligence in Greek teacher education: findings from a short intervention. Curr. Psychol. 42, 9282–9292. doi: 10.1007/s12144-021-02226-0

[ref73] Latif S. Amirullah M. (2020). Students’ academic resilience profiles based on gender and cohort. J. Kajian Bimbingan Konseling 5, 175–182. doi: 10.17977/um001v5i42020p175, PMID: 27261199

[ref74] Lazarides R. Fauth B. Gaspard H. Göllner R. (2021). Teacher self-efficacy and enthusiasm: relations to changes in student-perceived teaching quality at the beginning of secondary education. Learn. Instruct. 73:101435. doi: 10.1016/j.learninstruc.2020.101435

[ref75] Lazarides R. Schiefele U. Hettinger K. Frommelt M. C. (2023). Tracing the signal from teachers to students: how teachers’ motivational beliefs longitudinally relate to student interest through student-reported teaching practices. J. Educ. Psychol. 115, 290–308. doi: 10.1037/edu0000777

[ref76] Lazarus R. Folkman S. (1986). Estrés y procesos cognitivos. Barcelona: Martínez Roca.

[ref77] Leonardi F. Astuti N. W. (2023). Hubungan stres kerja dengan kesejahteraan psikologis guru. Provitae J. Psikologi Pendidikan 16, 26–37. doi: 10.24912/provitae.v16i2.26700

[ref78] Li M. Jones B. D. Williams T. O. Guo Y. J. (2022). Chinese students' perceptions of the motivational climate in college english courses: relationships between course perceptions, engagement, and achievement. Front. Psychol. 13:853221. doi: 10.3389/fpsyg.2022.853221, PMID: 35677126 PMC9169984

[ref79] Liang J. T. (2024). Developing emotional intelligence in a static and interactive music learning environment. Front. Psychol. 15:1279530. doi: 10.3389/Fpsyg.2024.1279530 38375118 PMC10875094

[ref80] Liang W. Y. Song H. Sun R. (2022). Can a professional learning community facilitate teacher well-being in China? The mediating role of teaching self-efficacy. Educ. Stud. 48, 358–377. doi: 10.1080/03055698.2020.1755953

[ref81] Liu L. Fathi J. Allahveysi S. P. Kamran K. (2023). A model of teachers' growth mindset, teaching enjoyment, work engagement, and teacher grit among EFL teachers. Front. Psychol. 14:1137357. doi: 10.3389/Fpsyg.2023.1137357 36968701 PMC10030517

[ref82] Liu S. Onwuegbuzie A. J. (2012). Chinese teachers’ work stress and their turnover intention. Int. J. Educ. Res. 53, 160–170. doi: 10.1016/j.ijer.2012.03.006, PMID: 38566801

[ref83] Lucas-Mangas S. Valdivieso-León L. Espinoza-Díaz I. M. Tous-Pallarés J. (2022). Emotional intelligence, psychological well-being and burnout of active and in-training teachers. Int. J. Environ. Res. Public Health 19:3514. doi: 10.3390/ijerph19063514, PMID: 35329207 PMC8951300

[ref84] Maamari B. E. Salloum Y. N. (2023). The effect of high emotionally intelligent teachers on their teaching effectiveness at universities: the moderating effect of personality traits. Int.l J. Educ. Manag. 37, 575–590. doi: 10.1108/Ijem-12-2020-0565

[ref85] Madhukar G. (2021). A study on emotional intelligence of school teachers in Secunderabad. Int. J. Current Res. Rev. 13, 147–151. doi: 10.31782/IJCRR.2021.132125

[ref86] Maffoni S. I. Kalmpourtzidou Α. Cena H. (2021). The potential role of nutrition in mitigating the psychological impact of COVID-19 in healthcare workers. NFS J. 22, 6–8. doi: 10.1016/j.nfs.2020.12.002 PMC7833967 40477331

[ref87] Maharaj P. Ramsaroop A. (2022). Emotional intelligence as a contributor to enhancing educators' quality of life in the COVID-19 era. Front. Psychol. 13:921343. doi: 10.3389/fpsyg.2022.921343, PMID: 36072055 PMC9443812

[ref88] Mancini G. Mameli C. Biolcati R. (2022). Burnout in italian primary teachers: the predictive effects of trait emotional intelligence, trait anxiety, and job instability. Eur. J. Psychol. 18, 168–180. doi: 10.5964/ejop.2685, PMID: 36348695 PMC9632545

[ref89] Manzano-García G. Ayala J. C. (2013). Psychometric properties of Connor-Davidson resilience scale in a Spanish sample of entrepreneurs. Psicothema 25, 245–251. doi: 10.7334/psicothema2012.183, PMID: 23628541

[ref90] Mao Y. H. Luo X. Y. Wang S. J. Mao Z. Z. Xie M. Bonaiuto M. (2024). Flow experience fosters university students’ well-being through psychological resilience: a longitudinal design with cross-lagged analysis. British J. Educ. Psychol 94, 518–538. doi: 10.1111/bjep.12661, PMID: 38238106

[ref91] Marco-Ahulló A. Villarrasa-Sapiña I. Monfort-Torres G. (2022). Estudio descriptivo sobre las diferencias de género en el estrés académico derivado del contexto COVID-19 en población universitaria española. Retos Nuevas Tend. Educ. Física, Deporte Recreación 43, 845–851.

[ref92] Masková I. (2023). Work-related coping behaviour and experience patterns in university students: a review of 20 years of research. Front. Psychol. 14:1062749. doi: 10.3389/Fpsyg.2023.1062749 37143596 PMC10151672

[ref93] Maslach C. Jackson S. E. Leiter M. P. Schaufeli W. B. Schwab R. L. (1986). Maslach burnout inventory. Washington: Consulting Psychologists Press.

[ref94] Maslach C. Leiter M. P. (2022). The burnout challenge: managing People’s relationships with their jobs. Cambridge: Harvard University Press.

[ref95] Matos M. D. M. Iaochite R. T. Sharp J. G. (2022). Lecturer self-efficacy beliefs: an integrative review and synthesis of relevant literature. J. Further Higher Educ. 46, 225–245. doi: 10.1080/0309877X.2021.1905155

[ref96] Matud M. P. López-Curbelo M. Fortes D. (2019). Gender and psychological well-being. Int. J. Environ. Res. Public Health 16:3531. doi: 10.3390/ijerph16193531, PMID: 31547223 PMC6801582

[ref97] Mat-Zin N. I. Zainudin Z. N. Sulong R. M. (2023). Relationship between resilience and school culture with psychological well-being of school teachers. Int. J. Academic Res. Bus. Soc. Sci. 13, 5291–5301. doi: 10.6007/IJARBSS/v13-i12/20381

[ref98] Mayer J. D. Salovey P. (1993). The intelligence of emotional intelligence. Intelligence 17, 433–442. doi: 10.1016/0160-2896(93)90010-3, PMID: 39215247

[ref99] Mayer J. D. Salovey P. (1997). “What is emotional intelligence?” in emotional development and emotional intelligence: Educational implications. eds. Salovey P. Sluyter D. J. (Nueva York: Basic Books), 3–34.

[ref100] McKenzie J. Olson R. E. Patulny R. Bellocchi A. Mills K. A. (2019). Emotion management and solidarity in the workplace: a call for a new research agenda. Sociolo. Rev. 67, 672–688. doi: 10.1177/0038026118822982

[ref101] Mclean L. Bryce C. Johnson B. (2023a). Describing teachers' well-being prior to and 18 months after COVID-19 school closures, with a focus on early-career teachers and teachers of color. SAGE Open 13, 1–15. doi: 10.1177/21582440231217872

[ref102] Mclean L. Granger K. L. Chow J. C. (2023b). Associations between elementary teachers' mental health and students' engagement across content areas. Contem. Educ. Psychol. 75:102231. doi: 10.1016/j.cedpsych.2023.102231

[ref103] Mena M. A. Ruiz A. M. Vargas A. D. P. V. (2023). Diferencias de género en la percepción de estrés en universitarios del Ecuador. Ciencia Latina Rev. Cient. Multidisciplinar 7, 2026–2038. doi: 10.37811/cl_rcm.v6i6.3850

[ref104] Mennes H. von der Embse N. Kim E. Sundar P. Hines D. Welliver M. (2023). Are “well” teachers “better” teachers? A look into the relationship between first-year teacher emotion and use of evidence-based instructional strategies. Sch. Psychol. 39, 325–335. doi: 10.1037/spq0000593 37956072

[ref105] Mercader-Rubio I. Gutiérrez Ángel N. Oropesa Ruiz N. F. Sánchez-López P. (2022). Emotional intelligence, interpersonal relationships and the role of gender in student athletes. Int. J. Environ. Res. Public Health 19:9212. doi: 10.3390/ijerph19159212, PMID: 35954568 PMC9368593

[ref106] Miles J. A. Naumann S. E. (2023). Gender differences in intentions to seek personal counselling: the mediating role of social self-concept. British J. Guid. Couns., 52, 1–13. doi: 10.1080/03069885.2023.2196711

[ref107] Mugambi M. M. Mwove D. K. Musalia F. G. (2015). Influence of teachers on classroom learning environment. J. Educ. Policy Entrepreneurial Res. 2, 90–102.

[ref108] Navas-Casado M. L. García-Sancho E. Salguero J. M. (2023). Associations between maladaptive and adaptive emotion regulation strategies and aggressive behavior: a systematic review. Aggres. Violent Behav. 71:101845. doi: 10.1016/j.avb.2023.101845

[ref109] Nazir N. Zamir S. Bibi A. (2021). Gender based analysis of psychological well-being among university teachers. Global Educ. Studies Rev. 6, 243–252. doi: 10.31703/gesr.2021(VI-I).25

[ref110] Nemiro A. Hijazi Z. O'Connell R. Coetzee A. Snider L. (2022). Mental health and psychosocial wellbeing in education: the case to integrate core actions and interventions into learning environments. Mental Health Psychos. Work Counsel. Areas Armed Conflict 20, 36–45. doi: 10.4103/intv.intv_20_21

[ref111] Ng E. C. Fisher A. T. (2013). Understanding well-being in multi-levels: a review. Health Cult. Soc. 5, 308–323. doi: 10.5195/HCS.2013.142, PMID: 37483043

[ref112] Orellana G. A. (2022). Burnout y bienestar psicológico en docentes de tres colegios privados de la ciudad de Guatemala. Perspec. Propuestas Inv. Psicol. 4, 39–51. doi: 10.36631/RPH.2022.04.03

[ref113] Pajares F. Schunk D. H. (2002). “Self and self-belief in psychology and education: a historical perspective” in Improving academic achievement. ed. Aronson J. (United States: Academic Press), 3–21.

[ref114] Pei Y. Han J. Zhao J. Liu M. Pang W. (2022). The effects of the creator’s situation on creativity evaluation: the rater’s cognitive empathy and affective empathy matter in rating creative works. J. Intelligence 10:75. doi: 10.3390/jintelligence10040075, PMID: 36278597 PMC9590041

[ref115] Pérez J. M. Dorado A. Rodríguez-Brioso M. D. M. López J. (2020). Resiliencia para la promoción de la salud en la crisis Covid-19 en España. Rev. Ciencias Sociales 26, 52–63.

[ref116] Pérez A. B. D. Quispe F. M. P. Aguilar O. A. G. Cortez L. C. C. (2020). Transición secundaria-universidad y la adaptación a la vida universitaria. Rev. Ciencias Soc. 26, 244–258.

[ref117] Poormahmood A. Moayedi F. Alizadeh K. H. (2017). Relationships between psychological well-being, happiness and perceived occupational stress among primary school teachers. Arch. Hellenic Med. 34, 504–510.

[ref118] Pozo-Rico T. Gilar-Corbi R. Izquierdo A. Castejón J.-L. (2020). Teacher training can make a difference: tools to overcome the impact of COVID-19 on primary schools. An experimental study. Int. J. Environ. Res. Public Health 17:8633. doi: 10.3390/ijerph17228633, PMID: 33233750 PMC7699930

[ref119] Pozo-Rico T. Poveda R. Gutiérrez-Fresneda R. Castejón J.-L. Gilar-Corbi R. (2023). Revamping teacher training for challenging times: teachers’ well-being, resilience, emotional intelligence, and innovative methodologies as key teaching competencies. Psychol. Res. Behav. Manag. 16, 1–18. doi: 10.2147/Prbm.S382572 36636290 PMC9830420

[ref120] Pozo-Rico T. Sánchez-Sánchez B. Castejón J.-L. Gilar-Corbi R. (2018). Training course on emotional intelligence: the experience of emotional intelligence in a secondary education project. Publica 48, 235–255. doi: 10.30827/publicaciones.v48i2.8342

[ref121] Pozo-Rico T. Sandoval I. (2020). Can academic achievement in primary school students be improved through teacher training on emotional intelligence as a key academic competency? Front. Psychol. 10:2976. doi: 10.3389/Fpsyg.2019.02976 31998203 PMC6967735

[ref122] Procópio L. F. Pereira A. Procópio M. (2016). Fontes de stress em docentes: Estudo psicométrico sobre stress no estágio pedagógico. Revista EDaPECI 16, 455–468. doi: 10.29276/redapeci.2016.16.35965.455-468

[ref123] Quintero S. Hernández J. A. (2021). Síntomas de depresión asociados al síndrome de burnout ya condiciones socio laborales de docentes de colegios públicos de Envigado (Colombia). Psicol. Caribe 38, 133–147. doi: 10.14482/psdc.38.1.158.724

[ref124] Rana A. Soodan V. (2019). Effect of occupational and personal stress on job satisfaction, burnout, and health: a cross-sectional analysis of college teachers in Punjab, India. Indian J. Occup. Environ. Medicine 23, 133–140. doi: 10.4103/ijoem.IJOEM_216_19, PMID: 31920263 PMC6941331

[ref125] Ratanasiripong P. Ratanasiripong N. T. Nungdanjark W. Thongthammarat Y. Toyama S. (2022). Mental health and burnout among teachers in Thailand. J. Health Res. 36, 404–416. doi: 10.1108/Jhr-05-2020-0181

[ref126] Razia B. (2016). Emotional intelligence of pupil teachers in relation to their well-being. Int. Res. J. Social Sciences 5, 20–23.

[ref9001] Rezaull k. Ahmed N. Taherun M. Nesa T. Billah M. Wienaah P. (2021). Self-Efficacy: A Key Components of Teacher Effectiveness. Asian J. Educ. Social Studies, 25, 24–34. doi: 10.9734/ajess/2021/v25i130590

[ref127] Regalado A. Mena J. Gratacós G. (2021). “Resilience as a crucial training topic in teacher induction plans: a systematic literature review” in Teacher induction and mentoring. Palgrave studies on leadership and learning in teacher education. eds. Mena J. Clarke A. (London: Palgrave Macmillan), 67–95.

[ref128] Rellon J. A. Quiambao V. G. Cerna M. G. Asuncion C. S. Baguio J. B. Kintanar G. N. (2024). A path model of psychological well-being of teachers during the covid–19 pandemic: a positivist and constructivist viewpoint. Asian J. Educ. Soc. Stud. 50, 127–141. doi: 10.9734/ajess/2024/v50i51347

[ref129] Remor E. (2006). Psychometric properties of a european spanish version of the perceived stress scale (PSS). Span. J. Psychol. 9, 86–93. doi: 10.1017/S1138741600006004, PMID: 16673626

[ref130] Roa L. A. B. Castillo F. B. Espino J. O. G. Quezada G. R. H. Cabrera U. O. I. de Arévalo D. P. (2023). Burnout Académico en Universidades Latinoamericanas. Editorial Mar Caribe de Josefrank Pernalete Lugo: Perú.

[ref131] Rodríguez-Sánchez A. Guinot J. Chiva R. López-Cabrales Á. (2021). How to emerge stronger: antecedents and consequences of organizational resilience. J. Manag. Organ. 27, 442–459. doi: 10.1017/jmo.2019.5

[ref132] Rojas-Solís J. L. Totolhua-Reyes B. A. Rodríguez-Vásquez D. J. (2021). Síndrome de burnout en docentes universitarios latinoamericanos: una revisión sistemática. Espiral. Cuadernos Prof. 14:657. doi: 10.25115/ecp.v14i29.4657

[ref133] Romano L. Consiglio P. Angelini G. Fiorilli C. (2021). Between academic resilience and burnout: the moderating role of satisfaction on school context relationships. Euro. J. Inv. Health Psychol. Educ. 11, 770–780. doi: 10.3390/ejihpe11030055, PMID: 34563068 PMC8314378

[ref134] Romano L. Tang X. Hietajärvi L. Salmela-Aro K. Fiorilli C. (2020). Students’ trait emotional intelligence and perceived teacher emotional support in preventing burnout: the moderating role of academic anxiety. Int. J. Environ. Res. Public Health 17:4771. doi: 10.3390/ijerph17134771, PMID: 32630744 PMC7369914

[ref135] Ryff C. D. (1989). Beyond Ponce de Leon and life satisfaction: new directions in quest of successful ageing. Int. J. Behav. Dev. 12, 35–55. doi: 10.1177/016502548901200102

[ref136] Sabater M. M. Mata J. C. (2019). Poder y diversidad. Los aportes de la Interseccionalidad a la didáctica de las ciencias sociales. CLIO. Hist. Hist. Teach. 45, 139–154. doi: 10.26754/ojs_clio/clio.2019458646

[ref137] Salami S. O. (2010). Occupational stress and well-being: emotional intelligence, self-efficacy, coping, negative affectivity and social support as moderators. J. Int. Social Res. 3, 387–398.

[ref138] Saldarriaga O. Ledesma M. J. Malpartida J. N. Diaz J. R. (2022). Resiliencia docente en las escuelas públicas de Lima Metropolitana – Perú. Rev. Ciencias Sociales 28, 261–274. doi: 10.31876/rcs.v28i1.37690

[ref139] Salovey P. Mayer J. D. Goldman S. L. Turvey C. Palfai T. P. (1995). “Emotional attention, clarity, and repair: exploring emotional intelligence using the trait meta-mood scale” in Emotion, disclosure, and health. ed. Pennebaker J. W. (Washington: American Psychological Association), 125–154.

[ref140] Sarrionandia A. Mikolajczak M. (2019). A meta-analysis of the possible behavioural and biological variables linking trait emotional intelligence to health. Health Psychol. Rev. 14, 220–244. doi: 10.1080/17437199.2019.1641423 31284846

[ref141] Schnaider-Levi L. Ganz A. B. Zafrani K. Goldman Z. Mitnik I. Rolnik B.. (2020). The effect of inquiry-based stress reduction on teacher burnout: a controlled trial. Brain Sci. 10:468. doi: 10.3390/brainsci10070468, PMID: 32708055 PMC7407507

[ref142] Seibt R. Kreuzfeld S. (2023). Working time reduction, mental health, and early retirement among part-time teachers at German upper secondary schools – a cross-sectional study. Front. Public Health 11:1293239. doi: 10.3389/Fpubh.2023.1293239 38074760 PMC10710235

[ref143] Seisdedos N. (1997). Maslach burnout inventory: burnout syndrome due to stress in the helping professions. Madrid: TEA Ediciones.

[ref144] Sigcha L. G. P. Gómez G. D. G. (2022). El estrés y su relación con la inteligencia emocional en docentes universitarios. Ciencia Latina Rev. Cient. Multidiscip. 6, 1357–1372. doi: 10.37811/cl_rcm.v6i3.2301

[ref145] Skaalvik E. M. Skaalvik S. (2007). Dimensions of teacher self-efficacy and relations with strain factors, perceived collective teacher efficacy, and teacher burnout. J. Educ. Psychol. 99, 611–625. doi: 10.1037/0022-0663.99.3.611

[ref146] Sojer P. Kainbacher S. Hüfner K. Kemmler G. Deisenhammer E. A. (2024). Trait emotional intelligence and resilience: gender differences among university students. Neuropsychiatrie 38, 39–46. doi: 10.1007/s40211-023-00484-x, PMID: 37982957

[ref147] Sommovigo V. Setti I. Argentero P. (2019). The role of service providers’ resilience in buffering the negative impact of customer incivility on service recovery performance. Sustain. For. 11:285. doi: 10.3390/su11010285

[ref148] Sönmez S. Kolaşınlı I. B. (2021). The effect of preschool teachers’ stress states on classroom climate. Int. J. Prim. Elem. Early Years Educ. 49, 190–202. doi: 10.1080/03004279.2019.1709528

[ref149] Sousa D. Barros C. (2017). Being a teacher in the current context of work: psychosocial risks and consequences for health and well-being. Int. J. Work. Cond. 14, 17–32.

[ref150] Suárez-Martel M. J. Santana J. D. (2021). The mediating effect of university teaching staff’s psychological well-being between emotional intelligence and burnout. Psicol. Educ. 27, 145–153. doi: 10.5093/psed2021a12

[ref151] Suleyeva K. Tovma N. Zakirova O. (2022). Developing emotional intelligence in elementary school children in Russia: verbal and non-verbal communication. Int. J. Prim. Elem. Early Years Educ. 50, 1095–1106. doi: 10.1080/03004279.2021.1934060

[ref152] Supervía P. U. Bordás C. S. (2020). Burnout syndrome, engagement and goal orientation in teachers from different educational stages. Sustain. For. 12:6882. doi: 10.3390/su12176882

[ref153] Tejasree S. (2019). A study on stress management at Sai Sanjeevini hospitals. Int. J. Res. Applied Science Engin. Tech. 7, 444–448. doi: 10.22214/ijraset.2019.9062

[ref154] Téllez-Martínez S. Cantón-Mayo I. García-Martín S. (2021). Impedimentos a la consecución de la satisfacción y el bienestar docente. Campus Virtuales 10, 185–193.

[ref155] Terrell K. R. Boggs C. Adelstein D. Hamadi H. Y. Kulikov E. Martinez V.. (2023). Mental health initiatives: providing stress management, wellness, and mindfulness workshops on college campuses. J. American College Health 8, 1–8. doi: 10.1080/07448481.2023.2222830 37290001

[ref156] Tikhomirova M. A. Bordovskaia N. V. Koshkina E. A. (2022). Professional and personal determinants of teachers' psychological well-being. Educ. Self Dev. 17, 188–202. doi: 10.26907/esd.17.2.15, PMID: 39192279

[ref157] Toral C. K. H. Mera M. V. V. Gracia N. S. G. Arroyo H. G. (2023). Cansancio mental y su repercusión en el desempeño académico. Ibero Amer. J. Educ. Soc. Res. 3, 95–104. doi: 10.56183/iberoeds.v3i2.639

[ref158] Tribhuvan S. L. (2020). A study of stress and psychological well-being among senior college teachers. Int. J. Indian Psychol. 8, 78–82. doi: 10.25215/0804.012

[ref159] Ulla T. Poom-Valickis K. (2023). Program support matters: a systematic review on teacher-and school related contextual factors facilitating the implementation of social-emotional learning programs. Front. Educ. 7:965538. doi: 10.3389/feduc.2022.965538

[ref160] Vandenberghe R. Huberman A. (1999). Understanding and preventing teacher burnout. New York: Cambridge University Press.

[ref161] Vargas W. C. García M. (2021). Resiliencia, comprensión psicosocial para los pospenados del Instituto Nacional Penitenciario y Carcelario en Colombia. Rev. Ciencias Sociales 27, 151–167. doi: 10.31876/rcs.v27i.36499

[ref162] Vazi M. L. Ruiter R. A. Van den Borne B. Martin G. Dumont K. Reddy P. S. (2013). The relationship between wellbeing indicators and teacher psychological stress in eastern cape public schools in South Africa. SA J. Ind. Psychol. 39, 1–10. doi: 10.4102/sajip.v39i1.1042

[ref163] Veloso-Besio C. Cuadra-Peralta A. Antezana-Saguez I. Avendaño-Robledo R. Fuentes-Soto L. (2013). Relación entre inteligencia emocional, satisfacción vital, felicidad subjetiva y resiliencia en funcionarios de educación especial. Est. Pedagóg. 39, 355–366. doi: 10.4067/S0718-07052013000200022

[ref164] Wang X. (2023). Exploring positive teacher-student relationships: the synergy of teacher mindfulness and emotional intelligence. Front. Psychol. 14:1301786. doi: 10.3389/Fpsyg.2023.1301786 38094701 PMC10716249

[ref165] Wang P. Chu P. Wang J. Pan R. Sun Y. Yan M.. (2020). Association between job stress and organizational commitment in three types of Chinese university teachers: mediating effects of job burnout and job satisfaction. Front. Psychol. 11:576768. doi: 10.3389/fpsyg.2020.576768, PMID: 33132985 PMC7578428

[ref166] Wang Y. B. Wang Y. Y. (2022). The interrelationship between emotional intelligence, self-efficacy, and burnout among foreign language teachers: a meta-analytic review. Front. Psychol. 13:913638. doi: 10.3389/fpsyg.2022.913638, PMID: 35837628 PMC9274274

[ref167] West N. R. B. Wan A. Morán N. Polo D. Torres E. (2021). Influencia de los estresores académicos en los niveles de estrés de los estudiantes de la Facultad de Ingeniería Industrial pertenecientes a la Universidad Tecnológica de Panamá. Pris. Tecno. 12, 60–64. doi: 10.33412/pri.v12.1.2798

[ref168] Wilcoxen C. Bell J. Steiner A. (2020). Empowerment through induction: supporting the well-being of beginning teachers. Int. J. Mentor. Coach. Educ. 9, 52–70. doi: 10.1108/Ijmce-02-2019-0022

[ref169] Wilkins N. J. Verlenden J. M. V. Szucs L. E. Johns M. M. (2023). Classroom management and facilitation approaches that promote school connectedness. J. Sch. Health 93, 582–593. doi: 10.1111/josh.13279, PMID: 36464639 PMC10935603

[ref170] Wisudawati W. N. (2023). Descriptive study of teacher self efficacy with gender perspective in kindergarten teachers. J. Southeast Asia Psychol. 11, 65–77. doi: 10.51200/sapj.v11i1.4886

[ref171] Woolcott G. Whannell R. Marshman M. Galligan L. Yeigh T. Axelsen T. (2023). Exploring pre-service teachers' affective-reflective skills: the effect of variations of a novel self-evaluation protocol. Asia Pacific J. Teach. Educ. 52, 126–154. doi: 10.1080/1359866X.2023.2227942

[ref172] Yang X. B. Du J. (2024). The effect of teacher self-efficacy, online pedagogical and content knowledge, and emotion regulation on teacher digital burnout: a mediation model. BMC Psychol. 12:1–13. doi: 10.1186/S40359-024-01540-Z 38279159 PMC10811882

[ref173] Yang L. Lee J. C. K. Zhang D. Chen J. J. (2023). Examining the relationships among teaching assistants' self-efficacy, emotional well-being and job satisfaction. Teach. Teaching. doi: 10.1080/13540602.2023.2265825, PMID: 38362215

[ref174] Zeidner M. Matthews G. Roberts R. D. (2012). The emotional intelligence, health, and well-being nexus: what have we learned and what have we missed? Applied Psychol. Health Wellbeing 4, 1–30. doi: 10.1111/j.1758-0854.2011.01062.x, PMID: 26286968

[ref175] Zhang S. Luo Y. Z. (2023). Review on the conceptual framework of teacher resilience. Front. Psychol. 14:1179984. doi: 10.3389/Fpsyg.2023.1179984 37546476 PMC10398336

